# Latency after Preterm Prelabor Rupture of the Membranes: Increased Risk for Periventricular Leukomalacia

**DOI:** 10.1155/2014/874984

**Published:** 2014-07-17

**Authors:** Annick Denzler, Tilo Burkhardt, Giancarlo Natalucci, Roland Zimmermann

**Affiliations:** ^1^Department of Obstetrics, Zurich University Hospital, Frauenklinikstraße 10, 8091 Zurich, Switzerland; ^2^Department of Neonatology, Zurich University Hospital, Frauenklinikstraße 10, 8091 Zurich, Switzerland

## Abstract

*Objective*. To identify the risk factors for cystic periventricular leukomalacia (cPVL) and their implications for deciding between immediate delivery and conservative management of preterm prelabor rupture of the membranes (pPROM). *Methods*. The following risk factors were compared between cPVL infants and 6440 controls: chorioamnionitis, sex, gestational age (GA), birth weight, pPROM, and pPROM-delivery interval. Factor impact on cPVL risk and clinical decision-making was determined by multivariate logistic regression. *Results*. Overall cPVL prevalence (*n* = 32) was 0.99/1000 births. All cPVL infants but one were born <34 weeks of gestation and were <2500 g; 56% had histological chorioamnionitis versus 1.1% of controls (OR 35.9; 95%-CI 12.6–102.7). Because chorioamnionitis is a postnatal diagnosis, logistic regression was performed with prenatally available factors: pPROM-delivery interval >48 hours (OR 9.0; 95%-CI 4.1–20.0), male gender (OR 3.2; 95%-CI 1.4–7.3). GA was not a risk factor if birth weight was included. Risk decreased with increasing fetal weight despite a prolonged pPROM-delivery interval. *Conclusion*. pPROM-delivery interval is the single most important prenatally available risk factor for the development of cPVL. Immediate delivery favors babies with chorioamnionitis but disfavors those with non infectious pPROM. In the absence of clinical chorioamnionitis fetal weight gain may offset the inflammatory risk of cPVL caused by a prolonged pPROM-delivery interval.

## 1. Introduction

Cerebral palsy includes a group of nonprogressive movement disorders due to brain lesions or abnormalities in early development [1]. Its prevalence of 2 per 1000 newborns overall rises to 77 per 1000 preterms born at below 28 0/7 weeks of gestation [2, 3]. A major cause is cystic periventricular leukomalacia (cPVL) comprising necrosis and subsequent cyst formation of the periventricular white matter: 60–100% of children with cPVL develop cerebral palsy [4–6]. Although the etiology and pathogenesis of cPVL remain unelucidated, several perinatal risk factors appear involved [7]. Birth asphyxia is no longer assumed the principal culprit [8].

Chorioamnionitis is thought to provoke a fetal inflammatory response syndrome associated with increased fetal cytokines that may lead to neonatal brain injury. Several studies indicate that the cytokines can themselves damage white matter without bacteremia being required [8–15]. An important predictor of chorioamnionitis is preterm prelabor rupture of membranes (pPROM) [16]. One-third of women with pPROM have positive amniotic fluid cultures [17]. Chorioamnionitis is quite common and often subclinical: fever and inflammatory marker elevation are rare in the early stages, making diagnosis difficult. Against this background the optimal management of pPROM remains unknown. The risks of prematurity from immediate delivery have to be balanced against those of ascending intrauterine infection and its probable consequences. Moreover subclinical chorioamnionitis is believed to cause pPROM. At a gestational age below 34 0/7 weeks, half the gynecologists in Australia and New Zealand preferred to induce labor, while the other half chose conservative management [18]. Several studies recommend an active management after 30 weeks [19, 20]. A Cochrane review from 2010 found no evidence about which strategy is favorable [21]. Despite a lack of randomized studies [22] new British and German guidelines advise active management before 34 weeks and active management between 34 and 36 weeks. Zurich University Hospital has hitherto favored conservative management, delaying delivery until clinically mandatory, on the grounds that the higher mortality and morbidity of newborns at lower gestational age are proven whereas the effect of increasing cPVL risk by prolonging pregnancy remains unknown. Only a prospective randomized trial can provide a definite answer. The more limited objectives of the present study were to identify the risk factors for PVL in the conservative pPROM management setting and determine whether prolonging gestation outweighs the risk of cPVL due to chorioamnionitis.

## 2. Materials and Methods

The study population comprised all babies with cPVL born in Zurich University Hospital's obstetric department between 1993 and 2008. Cranial ultrasound was obtained in infants with gestational age below 32 0/7 weeks or birth weight below 1500 g at days 1, 3, and 7 of life and repeated weekly until hospital discharge. cPVL was defined according to de Vries et al. [23]. All 6440 infants born between 2005 and 2007 and not affected by PVL served as controls.

During the study period women with premature contractions received tocolytic drugs (hexoprenaline only until 2001, nifedipine or hexoprenaline from 2002 to 2008) for 48 hours to allow lung maturation with 24 mg of betamethasone. Urinary tract infection or bacterial vaginosis was treated with antibiotics (co-amoxiclav or clindamycin). Steroids were repeated every 10 days until 2002. Since then, all women with threatened preterm delivery have received a single course of steroids. Tocolysis was maintained thereafter if contractions recurred after stopping tocolysis. Management of pPROM pregnancy was largely consistent with British Greentop Guideline no. 44 [17]. After pPROM co-amoxiclav was used until 2001 when it was changed to erythromycin [24], chorioamnionitis was monitored using blood tests (including leukocytes and C-reactive protein (CRP) 12 hourly for the first 48 hours), maternal temperature, and fetal heart rate. Clinical chorioamnionitis (≥3 of following markers: leukocytes > 20,000/*μ*L, CRP > 40 mg/dL, maternal temperature > 38°C, maternal tachycardia > 100 bpm, and fetal tachycardia > 160 bpm) was treated with antibiotics (co-amoxiclav) and prompt delivery. If the diagnosis was uncertain, delivery was deferred until chorioamnionitis became clinically obvious or delivery could be delayed no longer for other reasons. Diagnosis was based on placental histology, positive amniotic fluid cultures sampled at cesarean section, or clinical parameters.

Babies born below 25 0/7 weeks were excluded in both groups because in most instances neonatal care was restricted to comfort care. Infants with cPVL were monitored for long-term follow-up. Neurodevelopmental disability was classified according to Palisano et al. [25]. The risk factors recorded in both groups were chorioamnionitis, pPROM-delivery interval, gestational age at delivery, birth weight, gender, race, and parity.

All statistical analyses were performed with STATA 10 Statistics/Data Analysis Software (Stata Corporation, College Station, TX) using Pearson's *χ* ^2^ test for comparisons of frequencies and Wilcoxon's rank-sum test for group comparisons. Odds ratios (OR) with 95% confidence intervals (CI) were calculated. Subsequent to univariate analysis, multivariate logistic regression was performed to test the impact of factors such as chorioamnionitis, gestational age, birth weight, gender, and pPROM-delivery interval on the incidence of cPVL. The results were used to calculate the risks of developing cPVL at different fetal weights and pPROM-delivery intervals.

Given that the analysis was of anonymized data, the study was exempt from local institutional review board approval. Follow-up data of study preterms below 32 0/7 weeks were extracted from the prospective national database of the Swiss Neonatal Network & Follow-up Group. According to the recommendations of our Research Ethics Committee the investigators were obliged to inform parents about the scientific use of anonymized data. Parents had the right to refuse participation of their child.

## 3. Results

Between 1993 and 2008, 32,276 infants were born at Zurich University Hospital, including 6027 (18.7%) preterms (below 37 0/7 weeks); over the same period 32 cases of cPVL were recorded, representing an overall prevalence of 0.99‰ (Tables 1 and 2).

**Figure 1:**
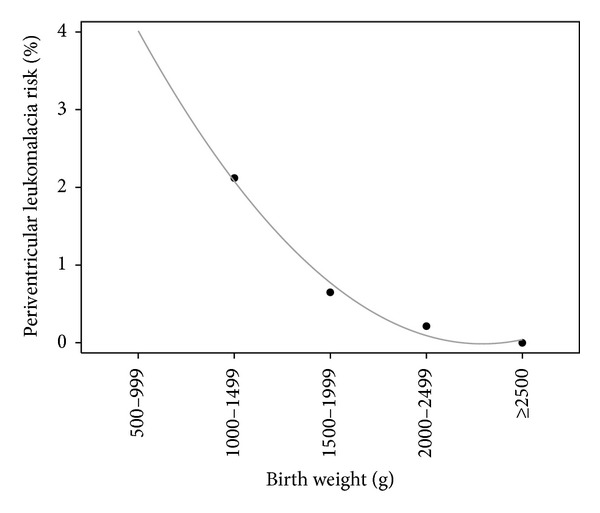
Exponential decrease in periventricular leukomalacia risk with increasing birth weight.

**Figure 2:**
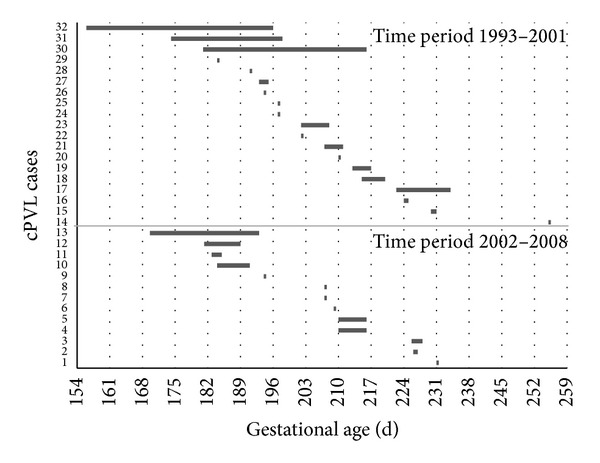
Data from 32 cPVL cases in two time periods. Each bar represents a pregnancy with pPROM-delivery interval beginning with gestational age of pPROM.

cPVL prevalence among preterm infants was 5.3‰. All 32 infants with PVL were delivered preterm and all but one before 34 0/7 weeks. cPVL risk decreased exponentially with increasing birth weight (Figure 1) and increasing gestational age. Males were 3 times more affected than females (male : female ratio 24 : 8). All birth weights in newborns with cPVL were less than 2500 g (Table 1). The individual pPROM-delivery intervals of all PVL cases are shown in Figure 2.

Median infant age at cPVL diagnosis was 19 days (3–40 days). Of the 32 infants, five (16%) died within the first 6 weeks after birth. Of the 27 surviving infants, three (11%) were lost to follow-up (parental refusal), while 24 (89%) were neurodevelopmentally assessed at a median (range) age of 3.8 years (2.0–10.2 years): one (4%) was normal, nine (38%) were moderately disabled, defined as cerebral palsy grade <3 according to the Gross Motor Function Classification System (GMFCS), or cognitive impairment with developmental quotient 55–69, or moderate visual or hearing impairment, and 14/24 (54%) were severely disabled, defined as disabling cerebral palsy (grade 3–5 GMFCS), or severe cognitive disability with developmental quotient <55, or major visual or hearing impairment.

Histologically confirmed chorioamnionitis was present in 16 cPVL infants (50%). A further two infants, for whom placental histology was missing, had ≥3 markers of clinical chorioamnionitis: leukocytes > 20,000/*μ*L, CRP > 40 mg/dL, maternal temperature > 38°C, maternal tachycardia > 100 bpm, and fetal tachycardia > 160 bpm. Thus, 18/32 cases (56%) were classified as having been complicated by chorioamnionitis. Two cases were assigned to the nonchorioamnionitis group despite the absence of placental histology and a number of clinical parameters.

The control group included 134 cases of suspected chorioamnionitis. Review of the placental histology and clinical parameters reduced these to 71 cases of histological chorioamnionitis and two cases of clinical chorioamnionitis in the absence of placental histology. Chorioamnionitis thus complicated 73/6440 (1.1%) of control deliveries.

Preliminary logistic regression revealed significant associations between cPVL and chorioamnionitis, male sex, and birth weight. Chorioamnionitis had the highest impact on cPVL risk (OR 35.9, 95% CI 12.6–102.7). However, because a prenatal diagnosis of chorioamnionitis is often not possible, logistic regression was performed, replacing chorioamnionitis by the pPROM-delivery interval. This revealed significant impacts on cPVL by sex (*P* = 0.008), pPROM-delivery interval > 48 hours (*P* < 0.001), and fetal weight (*P* < 0.001; Table 3).

Further multiple logistic regression analyses revealed significant associations between chorioamnionitis and pPROM-delivery interval > 24 hours (*P* = 0.002) and gestational age (*P* < 0.001). No significant influence of ethnicity (*P* = 0.49), fetal weight (*P* = 0.37), or parity (*P* = 0.79) was observed.

According to logistic regression analyses tabulation of estimated cPVL incidence at varying pPROM-delivery intervals and birth weights for boys and girls (Table 4), assuming fetal weight gain of 200 g/week [26], revealed a slight rise in the first 48 hours, followed by a significantly lower risk after the first and second week of prolongation of pregnancy. Increasing fetal weight during pPROM latency had a far stronger protective effect despite a prolonged pPROM-delivery interval being a risk factor for cPVL.

## 4. Discussion

Zurich University Hospital's obstetrics department is a tertiary referral center. This accounts for the high prevalence of preterm deliveries compared to the national average (19% versus 9%) [27]. The prevalence of cPVL in our study group (0.99‰) appears lower than the few reports in the literature. Hamrick et al. reported an incidence of 1.8% at UC San Francisco in 1992, falling to 0.2% in 2002; the incidence of cPVL in children weighing <1500 g decreased from 2.9% to 0.5% over the same period [28].

The difference may be partly due to Zurich's conservative management of newborns below 25 0/7 weeks of gestation (restriction to comfort care in the majority of cases) [29]. We may also have missed some cases of late cPVL diagnosis in children born after 32 0/7 weeks (there were no instances of late diagnosis of brain lesions in preterms included in long-term follow-up). Given our small sample size, we could only extrapolate cPVL incidence for birth weights <1000 g (Figure 1). The Vermont Oxford Network reported approximately 3% cPVL at birth weights 751–1500 g; risk was highest (6%) at birth weights <751 g [30]. Our data confirm the reported exponential decrease in cPVL incidence with increasing birth weight [28, 30]. They also confirm a similar exponential decrease with advancing gestational age independently of birth weight [30]. Again, our data at below 26 0/7 weeks are not comparable to other centers due to our conservative management of newborns around 25 0/7 weeks.

Our data support the dependency of cPVL risk on the pPROM-delivery interval [8, 31], but not on either low parity or PROM [8, 10, 31]. The finding of a 4 : 1 male/female ratio confirms previous reports [8, 15, 31] but remains unelucidated.

We also confirmed the several reports of a significant association between cPVL and chorioamnionitis [8, 9, 11, 14, 15]. The fact that only one cPVL baby had a positive blood culture within 3 days of birth supports the hypothesis that fetal inflammatory response syndrome is perfectly capable of causing brain damage even in the absence of bacteremia [32–34]. Apart from being a risk factor for cPVL, chorioamnionitis is well-recognized as correlating with neonatal morbidity and mortality [32–34].

Unfortunately, these risk factors cannot resolve our strategic dilemma of immediate versus delayed delivery for lowering short-term mortality and long-term sequelae. Chorioamnionitis is a major complication of pPROM but probably even more often the cause of pPROM.

The increase in cPVL risk during the first 48 hours after pPROM and the substantial decrease thereafter at varying birth weights and pPROM-delivery intervals (Table 4) can be interpreted in several ways. For example, the initial increase may relate to the use of antenatal steroids to induce lung maturation. Steroids could facilitate the spread of bacterial infection; they could also modulate the fetal inflammatory cytokines thought to cause brain damage [14]. Corroborative evidence is that the incidence of cPVL in our group decreased from 1.3‰ on repeated steroid courses to 0.7‰ on single-course steroids (although we must admit to having concomitantly switched to erythromycin and introduced nifedipine tocolysis). This study could not add any information about the impact of the different changed interventions to the lower cPVL incidence 2002–2008.

Another explanation is that the inverted U-shaped risk for cPVL with respect to the pPROM-delivery interval results from superposing two distinct groups of women: one with pPROM due to chorioamnionitis and the other with pPROM from a noninfectious cause. Most chorioamniotic pregnancies will deliver within a few days after pPROM. Thus, women still pregnant after one week of pPROM are more likely to have a noninfectious cause of fluid leakage. As a consequence, delaying delivery in these cases would lower cPVL risk by allowing birth weight to increase. At the same time, by increasing gestational age and birth weight, this strategy would substantially decrease all other complications of prematurity such as cerebral hemorrhage or lung pathology [35]. Conversely, a conservative strategy would increase cPVL risk in the infectious group. An increased risk for neurodevelopmental impairment in the first 48 to 72 hours after pPROM was also observed in a large French cohort with 1884 infants born at 24–32 weeks of gestational age [36].

Given this strong association between cPVL and chorioamnionitis, it is absolutely essential to diagnose intrauterine infection as early as possible. This points once again to the urgent need for a tool that reliably diagnoses chorioamnionitis.

In the absence of such a tool, either strategy carries a considerable risk of Pyrrhic victory. Because cPVL affects only a small proportion of newborns, with other problems of prematurity playing a much larger role, it seems not unreasonable to pursue a conservative strategy until prospective randomized trials provide a definitive answer or, at the least, until we have a reliable test for the early diagnosis of chorioamnionitis. Perinatal morbidity is strongly correlated with prematurity and latency does not appear to worsen outcome in pPROM [37].

The fact that chorioamnionitis and the pPROM-delivery interval seem to have a high impact on the risk of developing cPVL in our study could have important consequences for the future management of pPROM.

The strengths of this study are that all infants and mothers were monitored in the same department and that all preterms below 32 0/7 weeks received long-term follow-up, where possible (the actual follow-up rate was 89%). Only in midstudy were there any relevant changes in the management of pPROM (antibiotics, tocolytic, and steroid courses).

The study's limitations lie in its retrospective design and small cPVL sample size. We had particularly few birth weights below 1000 g, partly because the Swiss Society of Neonatology recommends restricting neonatal management to comfort care at gestational ages below 24 0/7 weeks. Between 24 0/7 and 25 6/7 weeks of gestation the decision to undertake intensive care is individual and influenced by prenatal factors such as birth weight, gender, antenatal steroid use, intrauterine growth restriction, chorioamnionitis, fetal malformation, multiple gestation, and clinical condition immediately after delivery (asphyxia, heart rate, activity, and response to initial resuscitation). Intervention continues in the neonatal intensive care unit with the primary goal of survival with an acceptable quality of life [29]. Our results are helpful for deliveries between 26 and 34 weeks of gestation only.

Other factors accounting for small sample size in any study of this kind include the fact that the true number of newborns with cPVL can be difficult to evaluate due to early postnatal death, especially when gestational age is very low. cPVL can often only be diagnosed weeks after birth because it takes time for the periventricular cysts to become visible on ultrasound. Failure of underreporting when making a late diagnosis of cPVL is another source.

Even if a prolonged pPROM-delivery interval may briefly increase the risk of cPVL, we believe that conservative management makes sense in the absence of clinical chorioamnionitis. Higher infant weight at delivery compensates for the impact of pPROM latency on neonatal outcome provided that the pregnancy can be prolonged by more than 48 hours.

## Figures and Tables

**Table 1:** Baseline cystic periventricular leukomalacia (cPVL) and control group characteristics.

Characteristic	cPVL (*n* = 32)	Controls (*n* = 6,440)	*P*
Gestational age (d)	207.5 (184–255)	273 (168–297)	<0.001
Below 34 gestational weeks (%)	97	9	<0.001
Multiparous (*n* [%])	12 (38)	3145 (49)	0.198
Birth weight (g, median [range])	1197.5 (740–2250)	3270 (300–5700)	<0.001
PROM (*n* [%])	18 (56)	714 (11)	<0.001
pPROM-delivery interval (h, median [range])	38 (0–960)	1.3 (0–2170)	0.004
Chorioamnionitis (*n* [%])	18 (56)	73 (1)	<0.001
Lung maturation administration (*n* [%])	27 (84)	281 (5)	<0.001
Infant sex (*n* [%])			
Female	8 (25)	3049 (47)	0.012
Male	24 (75)	3390 (53)	

PROM: premature rupture of the membranes; pPROM: preterm premature rupture of the membranes.

**Table 2:** Characteristics and management of the cystic periventricular leukomalacia (cPVL) group (*n* = 32) by study period.

Characteristic	Time period	Time period	Overall
	1993–2001	2002–2008	1993–2008
Chorioamnionitis			
Yes	12 (60)	6 (50)	18 (56)
No	6 (30)	6 (50)	12 (38)
Unknown	2 (10)	0 (0)	2 (6)
Premature rupture of membranes			
Yes	11 (55)	7 (58)	18 (56)
No	9 (45)	5 (42)	14 (44)
Antenatal betamethasone			
Yes	15 (75)	12 (100)	27 (84)
No	5 (25)	0 (0)	5 (16)
Infant sex			
Male	15 (75)	9 (75)	24 (75)
Female	5 (25)	3 (25)	8 (25)

Total	20 (1.3‰)	12 (0.7‰)	32 (0.95‰)

Data are *n* (%).

**Table 3:** Multivariate logistic regression analysis of the influence of fetal sex, preterm premature rupture of the membranes- (pPROM-) delivery interval, and birth weight on cystic periventricular leukomalacia (cPVL) prevalence.

Covariate	cPVL	*P*
Sex		
Female∗	1	
Male	3.1 (1.3–7.1)	0.008
pPROM-delivery interval		
≤48 h∗	1	
>48 h	8.2 (3.8–17.5)	<0.001
Per 100 g higher fetal weight	0.85 (0.81–0.89)	<0.001

Data are adjusted odds ratios (95% confidence intervals).

∗Baseline category.

**Table:** (a) Male infants

	Birth weight				
	500 g	1000 g	1500 g	2000 g	2500 g
pPROM-delivery interval					
0 h	0.268	0.096	0.035	0.012	0.004
24 h	0.279	0.100	0.036	0.013	0.005
48 h	0.291	0.105	0.038	0.014	0.005
1 week	0.240	0.086	0.031	0.012	0.004
2 weeks	0.214	0.077	0.028	0.010	0.004

**Table:** (b) Female infants

	Birth weight				
	500 g	1000 g	1500 g	2000 g	2500 g
pPROM-delivery interval					
0 h	0.081	0.029	0.010	0.004	0.001
24 h	0.085	0.030	0.011	0.004	0.001
48 h	0.088	0.032	0.011	0.004	0.001
1 week	0.073	0.026	0.009	0.003	0.001
2 weeks	0.065	0.023	0.008	0.003	0.001
